# What are the key points of bibliometrics?

**DOI:** 10.1097/JS9.0000000000001429

**Published:** 2024-04-04

**Authors:** Xincheng Li, Guosong Li, Jie Huang

**Affiliations:** a Department of Hepatobiliary Surgery, The Second Hospital of Baoshan City, Baoshan; b Department of Hepatopancreatobiliary Surgery, The Second Affiliated Hospital of Kunming Medical University, Kunming, People’s Republic of China

*Dear Editor,*

Cancers are highly intricate diseases characterized not only by the proliferation of malignant cells but also by an altered immune response. The modulation and reprogramming of the immune system play pivotal roles in tumor initiation and progression. Immunotherapy aims to reactivate anti-tumor immune cells and overcome the mechanisms through which tumors evade immune surveillance. The field of tumor immunotherapy has witnessed remarkable success in the clinic, primarily through the utilization of immune checkpoint blockade and adoptive cell transfer^1^. These approaches have demonstrated the ability to induce durable regression in certain refractory tumors that are unresponsive to conventional therapies. Notably, considerable research related to immunotherapy and tumor burden has been published, enhancing knowledge in this field. Additionally, it is facile to employ bibliometric methods to examine these myriad publications. Bibliometrics has provided us with a tool that can be easily scaled from the micro level (institute) to the macro level (world)^2^.

With great interest, we read the results of the review study conducted by Zhang and colleagues, recently published in the *International Journal of Surgery* ^3^. In this review article, Zhang *et al*. performed a bibliometric analysis of worldwide research trends on tumor burden and immunotherapy, indicating that the USA occupies an important position in article output and international collaborations. Future research hotspots, such as ‘nivolumab,’ ‘tumor microenvironment,’ and ‘immune checkpoint inhibitor,’ were identified. However, I would like to discuss some of my concerns.

Firstly, the description of the search strategy in this paper is not sufficiently accurate. The utmost importance of bibliometrics lies in employing an effective retrieval strategy to access a vast amount of literature, with the principle being to avoid omission and repetition^1^. However, the author’s search strategy in this article is overly simplistic, potentially resulting in overlooked literature and skewed results for bibliometric analysis. Similar to composing a review, it is imperative to elucidate the search strategy, which will facilitate subsequent follow-up and analysis of this trending subject matter. For instance, the search strategy employed for this article was established as follows: [topic search=(tumor burden OR tumor burden OR tumor load OR tumor load) AND (immunotherapy OR immunotherapies OR immunotherapeutic OR immunotherapeutics)]. However, when utilizing ‘tumor burden’ as the subject term in the PubMed MESH search, it was observed that the accurate search terms should also encompass: (Burden, Tumor OR Tumor Load OR Load, Tumor OR Loads, Tumor OR Tumor Loads OR Tumor Volume OR Volume, Tumor OR Tumor Weight OR Tumor Weights OR Weight, Tumor). The authors’ search terms for immunotherapy were limited to (immunotherapy OR immunotherapies OR immunotherapeutic OR immunotherapeutics). However, it is worth noting that they did not consider incorporating additional search terms related to immunotherapy into their search strategy: (immunotherapy OR immunotherapies OR immunotherapeutic OR immunotherapeutics OR ICI OR ICIs OR CPI OR immune-checkpoint inhibitor OR immune checkpoint inhibitor OR immune-checkpoint blockade OR immune checkpoint blockade OR Nivolumab OR pembrolizumab OR atezolizumab OR avelumab OR durvalumab OR ipilimumab OR PD-1OR cytotoxic T lymphocyte-associated antigen-4 OR CTLA-4).

Secondly, the existence of unclear, incorrectly spelled, abbreviated, and synonymous keywords has the potential to influence the results of both keyword co-occurrence analysis and hotspot analysis. Consequently, data cleaning becomes crucial in ensuring the accuracy of the outcomes. Data cleaning, alternatively referred to as data cleansing or scrubbing, entails the identification and elimination of errors and inconsistencies from the dataset, ultimately enhancing its quality^4^. Given the diverse manners of keyword usage, including the utilization of synonyms, abbreviations, and spelling errors, the presence of unclean keyword data may lead to misleading outcomes in co-occurrence analysis. However, the authors have neglected to discuss any measures related to data cleaning and sorting in their methodology section, or to provide an explanation on the implementation of quality control within the scope of literature retrieval.

Finally, in the section analyzing annual publication output results, the author mentioned an observed annual growth rate of 14.92%, yet failed to provide an explanation regarding the methodology employed for data calculation.

Despite these aforementioned concerns, this article potentially represents the first bibliometric analysis conducted within the field of tumor burden and immunotherapy. We express our gratitude to the author for their diligent review of articles pertaining to tumor burden and immunotherapy.

## Ethical approval

This paper is a correspondence and does not require ethical approval.

## Consent

This paper is a correspondence and does not require consent.

## Sources of funding

This work was supported by the Academic Leadership Training Program at the Second Affiliated Hospital of Kunming Medical University (Grant No. RCTDXS-202309) and the Kunming Medical University’s teaching research project (Grant No. 2023-JY-Y-059).

## Author contribution

X.L.: conceptualization, data curation, formal analysis, investigation, and methodology; G.L.: writing – original draft preparation; J.H.: data curation, supervision, and writing – reviewing and editing. All authors approved the final version of the manuscript.

## Conflicts of interest disclosure

There are no conflicts of interest.

## Research registration unique identifying number (UIN)

Not applicable.

## Guarantor

Jie Huang.

## Data availability statement

Data availability is not applicable to this article as no new data were created or analyzed in this study.

## Provenance and peer review

Not applicable.
